# Atlas and Epitome of Human Histology and Microscopic Anatomy

**Published:** 1904-03

**Authors:** 


### Reviews of Books.

**Atlas and Epitome of Human Histology and Microscopic Anatomy.** By Dr. JOHANNES SOBOTTA. Edited by CARL HUBER, M.D. Pp. 248. One hundred and seventy-one illustrations on eighty lithographic plates, and sixty-eight cuts in the text. Philadelphia and London: W. B. Saunders and Co. 1903.

To say that this is one of the best of Saunders's Hand Atlas Series is high praise. Being an illustrated text-book of histology with the technique omitted, it does not claim to be a laboratory hand-book. The text is in the American orthography, but readable and accurate; and the additions from the pen of the American editor are not unimportant. The great value of the book, however, rests upon the exceptionally fine lithographic plates. Since Klein and Noble Smith brought out their classic plates, nothing so good has been done in the way of a series of plates of normal histology. The colours are well chosen, and the registration in printing unusually accurate.

The majority of the subjects chosen are from the bodies of executed criminals obtained immediately after death—a welcome change from the sections of this, that, or the other from cats, rabbits or guinea-pigs, and of much greater use to the pathologist who wishes to refresh his memory of the human normal histology. In a book intended to teach normal histology to those entering the medical profession we require better and more illustrations of the cerebral cortex, the larynx and islands of Langerhans, resting mamma, etc. We also regret the entire omission of the names of the artists of whose pictures these plates are the reproductions.

The book is more remarkable from the very low price at which it is sold.

